# Factors influencing future perceptions of securing decent work among nursing students in South Korea: a cross-sectional descriptive study

**DOI:** 10.7586/jkbns.25.029

**Published:** 2025-08-22

**Authors:** Chung Hee Woo, Juah Kim

**Affiliations:** 1 College of Nursing, Konyang University, Deajeon, Korea; 2 Department of Nursing, Korean Bible University, Seoul, Korea

**Keywords:** Nursing, Work, Career choice, Perception, Professional role

## Abstract

**Purpose:**

This study aimed to identify the factors influencing nursing students' future perceptions of securing decent work by focusing on career adaptability, nursing professionalism, and social comparison orientation.

**Methods:**

This descriptive correlational study included 169 third- and fourth-year nursing students with clinical experience. Data were collected through an online survey conducted in October 2024 and analyzed using descriptive statistics, the t-test, one-way analysis of variance, Pearson correlation coefficients, and hierarchical multiple regression analysis with SPSS version 25.0.

**Results:**

Significant positive correlations were found between future perceptions of securing decent work and career adaptability (r = .35, *p* < .001) and nursing professionalism (r = .51, *p* < .001). However, social comparison orientation did not show a statistically significant relationship with future perceptions of securing decent work (r = −.09, *p* = .232). Hierarchical regression analysis revealed that intrinsic motivation, academic satisfaction, perceived economic constraints, and nursing professionalism significantly influenced future perceptions of securing decent work, explaining 45% of the variance.

**Conclusion:**

To improve nursing students' perceptions of securing decent work, it is necessary to foster a strong sense of nursing professionalism. Additionally, addressing perceived economic constraints and enhancing academic satisfaction can contribute to better preparedness for entering the professional workforce.

## INTRODUCTION

The era of the Fourth Industrial Revolution, characterized by rapid advancements in digital technology and artificial intelligence, has significantly altered existing jobs and the employment landscape [1]. The coronavirus disease 2019 (COVID-19) pandemic has led to job losses, increased unemployment rates, and shifts in work arrangements, such as remote working, thereby intensifying the societal perception of a need for a new normal regarding employment [2]. Decent work, as defined by the International Labour Organization (ILO), refers comprehensively to employment opportunities providing productive and fair income, workplace safety, social protection for families, better prospects for personal development and social integration, freedom of expression and organization, participation in decisions affecting one’s life, and guaranteed gender equality [2]. Although the ILO has emphasized that ensuring decent work in nursing requires addressing issues related to inadequate staffing and improvement of working hours [3], the COVID-19 pandemic has heightened threats to the safety and health of healthcare workers [4]. In South Korea, conflicts between the medical community and government have exacerbated employment instability among nurses and increased employment uncertainty for nursing students preparing to enter the profession [5]. Employment uncertainty negatively affects students preparing to enter the workforce by inducing adverse psychological states, causing a delay in decisions to engage in work, and reducing economic activity, thereby hindering their entry into decent jobs [6].

According to the Psychology of Working Theory (PWT), decent work includes a safe workplace, adequate rest and freedom, organizational values aligned with personal and community values, appropriate compensation, and healthcare benefits [7]. On the basis of PWT, work serves as a means of self-determination, encompassing career decisions influenced by both intrinsic and extrinsic motivations; hence, this theory provides a useful theoretical foundation for youth preparing to enter the workforce [8]. Initially, the primary target groups of PWT were marginalized populations, such as ethnic minorities and individuals with disabilities [7]; however, following the COVID-19 pandemic, the theory has expanded to include occupations involving face-to-face tasks that threaten personal safety, such as nursing [6]. In particular, since nursing students, in comparison to general college students, have concretely envisioned their future employment and occupation [9], it is appropriate to apply the decent work framework emphasized by the ILO and PWT while integrating a future-oriented perspective [10].

According to PWT, factors predicting decent work include work volition, career adaptability, economic constraints, and marginalization. Experiences of economic constraints and marginalization enhance decision-making abilities regarding quality career and career adaptability or preparedness, thereby influencing the attainment of quality employment [7]. However, since nursing curricula in South Korea are predominantly standardized because of the four-year education programs and nursing students’ socioeconomic constraints primarily stem from parental factors [9,10], it is effective to examine nursing students’ future perceptions of securing decent work by focusing on career adaptability [10]; nursing professionalism, which provides a conceptual framework for evaluating views and behaviors toward professional nursing [11]; and social comparison orientation [12], which can be activated during information-seeking from others in times of heightened employment uncertainty; while controlling for motivation for choosing a major and economic constraints.

Specifically, career adaptability refers to an individual’s self-regulation ability to cope with problems arising from occupational roles and career transitions [13]. Career adaptability is posited as a significant variable in securing decent work, as it either influences or moderates the relationship between individuals’ psychosocial characteristics and social contexts, such as employment conditions and unemployment rates [7]. International studies have demonstrated that career adaptability serves as a positive resource influencing job satisfaction among Chinese workers [14] and supporting Italian college students in managing career transitions [15]. Similarly, studies conducted in South Korea reported that career adaptability played a mediating role in college students’ abilities to overcome career barriers and engage in job preparation behaviors during the COVID-19 pandemic [16], as well as in nursing students’ capacities to overcome economic constraints and achieve positive future perceptions of securing decent work [9]. Nursing professionalism is an integrated perspective encompassing professional self-concept, social recognition, nursing expertise, roles in nursing practice, and nursing autonomy [17]. Considering nursing professionalism shapes nursing students’ beliefs and attitudes toward their future careers, subsequently influencing the quality of nursing services they provide upon entering clinical practice [18], it is anticipated to predict their future perceptions of securing decent work. Recently, nursing students have been experiencing significant employment uncertainty [5]. Perceptions of uncertainty facilitate external information-seeking activities, consequently increasing social comparison tendencies [19]. This individual tendency to compare oneself with others is defined as social comparison orientation [20]. As nursing students perceive greater employment uncertainty, social comparison orientation is more likely to be activated as part of efforts to reduce such uncertainty [12]. Therefore, it is meaningful to determine the extent to which social comparison orientation influences nursing students’ future perceptions of their careers as decent work. Hence, this study aims to investigate the effects of career adaptability, nursing professionalism, and social comparison orientation on nursing students’ future perceptions of securing decent work, providing foundational data to establish conditions for decent work in the nursing field.

## METHODS

### 1. Study design

This study employed a descriptive cross-sectional design to examine the influence of career adaptability, nursing professionalism, and social comparison orientation on nursing students’ future perceptions of securing decent work.

### 2. Participants

The dependent variable, future perceptions of securing decent work, was more suitable for students with concrete plans to enter the workforce [10]. For nursing students, clinical practicum experience is likely to have a direct influence on how they perceive their future profession [21]. Although the timing of the clinical practicum may vary across institutions, it typically begins no later than the third year of the nursing program. Therefore, study participants included third- and fourth-year nursing students. Using G*Power 3.1.9.4, the required sample size was calculated to be 160 for a regression analysis, based on a significance level of .05, power (1–β) of .95, a medium effect size (f^2^ = .15), and eight predictor variables (general participant characteristics and independent study variables). Considering an approximate dropout rate of 10%, 178 participants were recruited, and data from 169 participants were ultimately analyzed after excluding nine incomplete responses.

### 3. Instruments

The questionnaires utilized in this study comprised a total of 61 items: five items on general characteristics (sex, academic year, motivation for choosing a major, satisfaction with selected major, and economic constraints), 15 items assessing future perceptions of securing decent work, 12 items measuring career adaptability, 18 items measuring nursing professionalism, and 11 items assessing social comparison orientation.

#### 1) General characteristics

Based on previous studies, the general characteristics comprised five items: sex, academic year, motivation for choosing a major (internal and external motivation), satisfaction with selected major (satisfied, neutral, or dissatisfied), and perceived economic constraints (high, moderate, or low).

#### 2) Future perceptions of securing decent work

Future perceptions of securing decent work were measured using the Perceived Future Decent Work Securement Scale, originally developed for adults by Duffy et al. [7] and subsequently adapted and validated for Korean college students by Kim et al. [10]. Both the original scale [7] and the adapted scale of Kim et al. [10] consist of 15 items across five dimensions (physically and interpersonally safe working environment, healthcare support, adequate compensation, guaranteed leisure and rest, and organizational values aligned with family and community values). Each dimension is covered by three items measured on a 7-point Likert scale. To specifically reflect the future perspective of college students, items were adapted by Kim et al. [10] to explicitly reference future expectations. For example, the item “I receive adequate compensation for my work” was revised to “I will receive adequate compensation for my work in the future.” All adapted items were validated through confirmatory factor analysis. Responses were scored on a scale ranging from 1 (“Strongly disagree”) to 7 (“Strongly agree”), with higher scores indicating stronger expectations of securing decent work in the future. Cronbach’s α was .91 in Kim et al. [10], .89 in a study involving nursing students [9], and .85 in the current study.

#### 3) Career adaptability

Savickas and Porfeli [13] developed a 24-item instrument comprising four factors—concern about occupational future, control over one’s life and surroundings, curiosity about oneself and career opportunities, and confidence in completing tasks and overcoming obstacles—to measure career adaptability. In this study, career adaptability was assessed using the Career Adapt-Abilities Scale–Short Form, an abridged 12-item version (three items per factor) that was developed by Maggiori et al. [22] while preserving the original four-factor structure, and validated in Korean by Kim and Koh [23]. This instrument uses a 5-point Likert scale ranging from 1 (“Strongly disagree”) to 5 (“Strongly agree”), with higher scores indicating greater career adaptability. Cronbach’s α was .90 for both the German and French versions in Maggiori et al. [22], .89 in Kim and Koh [23], and .90 in the current study.

#### 4) Nursing professionalism

Nursing professionalism is defined as a systematic view of nursing as a profession, which encompasses nursing activities and occupational consciousness regarding nursing roles [11]. This study measured nursing professionalism using an instrument originally developed by Yeun et al. [17] and subsequently abbreviated and validated by Han et al. [24]. This instrument comprises 18 items across five factors: professional self-concept (six items), social recognition (five items), nursing expertise (three items), nursing roles (two items), and nursing autonomy (two items). Responses are scored on a 5-point Likert scale ranging from 1 (“Strongly disagree”) to 5 (“Strongly agree”), with higher scores indicating greater nursing professionalism. Cronbach’s α was .94 in Han et al. [24] and .85 in the current study.

#### 5) Social comparison orientation

Social comparison orientation was assessed using the Iowa-Netherlands Comparison Orientation Measure, originally developed by Gibbons and Buunk [20] and validated in Korean by Choi [25]. This instrument comprises 11 items categorized into two factors, ability (six items) and opinion (five items), and measured on a 5-point Likert scale. Responses range from 1 (“Strongly disagree”) to 5 (“Strongly agree”), with higher scores indicating greater social comparison orientation. Cronbach’s α was .83 in both Choi [25] and the present study.

### 4. Data collection

Data were collected from October 11 to October 30, 2024. The survey was distributed through online nursing student communities (e.g., Nursenote, Nurscape and Nurselife) by research assistants who had access to these communities. Distribution was in accordance with the communities’ respective posting guidelines. Participants who expressed interest, provided their contact information, and met the inclusion criteria were contacted, and the survey was administered using a professional online survey platform (Moaform). At the beginning of the survey, participants were asked to confirm whether they were enrolled in the third or fourth year of a college nursing program. Those who responded affirmatively were screened for additional eligibility criteria and provided with a study information sheet to inform their decision on whether they would participate. Informed consent was obtained online, and only participants who provided consent gained access to the survey items. Survey completion required approximately 10 minutes, and participants received a small token of appreciation upon completion. After excluding nine incomplete responses, data from a total of 169 respondents were included in the final analysis.

### 5. Data analysis

The collected data were analyzed using SPSS version 25.0 (IBM Corp., Armonk, NY, USA). Descriptive statistics were used to examine the participants’ general characteristics and properties of the study variables. The normality of each variable was assessed based on skewness and kurtosis. Differences in future perceptions of securing decent work according to participants’ general characteristics were analyzed using independent t-tests and analysis of variance, with Scheffé tests conducted for post-hoc analysis. Pearson correlation coefficients were computed to identify correlations among future perceptions of securing decent work, career adaptability, nursing professionalism, and social comparison orientation. Hierarchical multiple regression analysis was conducted to identify factors influencing participants’ future perceptions of securing decent work.

### 6. Ethical considerations

This study was conducted with approval from the Institutional Review Board of Konyang university (No. KYU 2024-09-005). Participants were provided with written information detailing the purpose of the study, their rights to refuse or withdraw participation, and privacy protections, and they provided written informed consent before commencing the survey. Before data collection, participants were presented with a clear on-screen option to indicate consent or non-consent after reviewing a comprehensive study information sheet. If consent was provided, the survey became accessible; if consent was denied, the survey automatically closed, and participation ceased. Participants were informed they could withdraw from the study at any point during participation. In case of withdrawal, all personal information and collected data were destroyed. Participants were assured there would be no penalties for withdrawal, and anonymity and confidentiality were guaranteed through coding procedures detailed in the provided information sheet.

## RESULTS

### 1. Participants’ general characteristics

Participants’ general characteristics included sex (men: n =34, 20.1%; women: n = 135, 79.9%) and academic year (third-year: n = 69, 40.8%; fourth-year: n = 100, 59.2%). Motivation for choosing a major was almost evenly distributed between internal (n = 85, 50.3%) and external motivation (n = 84, 49.7%). Regarding satisfaction with selected major, more than half the respondents were satisfied (n = 107, 63.3%), followed by neutral (n = 50, 29.6%) and dissatisfied (n = 12, 7.1%). Participants reporting low perceived economic constraints (n = 77, 45.6%) outnumbered those reporting high (n = 45, 26.6%) or moderate constraints (n = 47, 27.8%) (Table 1).

### 2. Levels of future perceptions of securing decent work, career adaptability, nursing professionalism, and social comparison orientation

Levels of participants’ future perceptions of securing decent work, career adaptability, nursing professionalism, and social comparison orientation are summarized in Table 2. Future perceptions of securing decent work had an average score of 4.47 ± 0.77 (on a 7-point scale). Career adaptability averaged 4.01 ± 0.45; nursing professionalism, 3.96 ± 0.43; and social comparison orientation, 3.68 ± 0.55 (each on a 5-point scale). Normality tests indicated absolute kurtosis values ranging from 0.08 to 0.38 (below 10) and absolute skewness values ranging from 0.17 to 0.37 (below 3), confirming the data were normally distributed.

### 3. Differences in future perceptions of securing decent work according to general characteristics

Analysis of differences in future perceptions of securing decent work according to general characteristics revealed statistically significant differences based on motivation for choosing a major (t = 3.62, *p* < .001), satisfaction with selected major (t = 26.22, *p* < .001), and perceived economic constraints (t = 6.82, *p* = .001). Post-hoc analysis indicated that the group with high satisfaction with their major showed statistically significant differences in comparison to the neutral or dissatisfied groups. Similarly, groups perceiving low economic constraints showed statistically significant differences in comparison to groups perceiving high economic constraints. No statistically significant differences were found according to sex (t = 1.43, *p* = .154) or academic year (t = −0.76, *p* = .448) (Table 3).

### 4. Correlations among future perceptions of securing decent work, career adaptability, nursing professionalism, and social comparison orientation

Correlation analysis among participants’ future perceptions of securing decent work, career adaptability, nursing professionalism, and social comparison orientation revealed statistically significant positive correlations between future perceptions of decent work and both career adaptability (r = .35, *p* < .001) and nursing professionalism (r = .51, *p* < .001). Career adaptability and nursing professionalism also demonstrated a statistically significant positive correlation (r = .43, *p* < .001). However, social comparison orientation showed no statistically significant correlation with future perceptions of decent work (r = −.09, *p* = .232), career adaptability (r = .03, *p* = .659), or nursing professionalism (r = .14, *p* = .081) (Table 4).

### 5. Factors influencing future perceptions of securing decent work

Hierarchical multiple regression analysis was conducted to identify factors influencing participants’ future perceptions of securing decent work. In Model 1, the general characteristics that exhibited differences in future perceptions of securing decent work (motivation for choosing a major, satisfaction with selected major, and perceived economic constraints) were entered as dummy variables. In Model 2, career adaptability and nursing professionalism, which were significantly associated with future perceptions of securing decent work in correlation and simple regression analyses, were additionally included in the regression model. The Durbin-Watson statistic was 2.03, indicating no autocorrelation issues. The Variance Inflation Factor ranged from 1.08 to 1.44 (below 10), and tolerance values ranged from .69 to .93 (above 0.1), confirming the suitability of the data for regression analysis.

Regression analysis revealed that Model 1 was statistically significant (F = 15.86, *p* < .001). Specifically, external motivation for choosing a major in comparison to internal motivation (β = −.18, *p* = .007), high satisfaction with selected major in comparison to neutral satisfaction (β = .44, p < .001), and high perceived economic constraints in comparison to low perceived constraints (β = −.26, *p* < .001) were identified as significant predictors. In Model 2, after controlling for the independent variables of Model 1, career adaptability and nursing professionalism were additionally included to assess their impact on future perceptions of securing decent work. Model 2 was statistically significant (F = 20.41, *p* < .001). The significant variables in Model 1 along with nursing professionalism (β = .38, *p* < .001) emerged as significant predictors influencing future perceptions of securing decent work. The explanatory power for future perceptions of securing decent work increased from 31% in Model 1 to 45% in Model 2 (Table 5).

## DISCUSSION

This study, grounded in the PWT, aimed to provide foundational data for establishing decent working conditions in the nursing field by examining the influences of career adaptability, nursing professionalism, and social comparison orientation on nursing students’ future perceptions of securing decent work.

In this study, third- and fourth-year nursing students’ mean score for future perceptions of securing decent work was 4.47 out of 7, lower than the scores reported for both first- to fourth-year nursing students (4.81) [9] and general college students in South Korea and the United States (5.15) [26]. These relatively lower scores for future perceptions of securing decent work among nursing students (compared with scores in previous studies) may result from heightened employment uncertainty due to prolonged conflicts between the government and medical sector, leading to substantial reductions in nurse recruitment [5]. Persistent chronic staffing shortages and ongoing demands for improved working conditions in nursing [3], combined with rising occupational uncertainty driven by external environmental changes, may increase career anxiety among job seekers [1]. Negative employment perceptions have been linked to unfavorable views of both job quantity and quality [1]. Therefore, efforts to expand nursing infrastructure and enhance working conditions may positively influence future perceptions of employment in nursing.

Analysis of differences in future perceptions of securing decent work according to participant characteristics revealed statistically significant differences based on motivation for choosing a major, satisfaction with selected major, and economic constraints. Regarding motivation for choosing a major, nursing students who chose their major based on intrinsic motivations, such as personal aptitude and interests, reported more positive perceptions of nursing careers than those influenced by extrinsic motivations, such as recommendations from family or friends or anticipation of high employment rates. Additionally, participants reporting greater satisfaction with their major exhibited more positive future perceptions of their career. This finding aligns with previous research demonstrating a positive correlation between nursing students’ favorable perceptions of their major and both career respect and career preparation behaviors [27]. Given the significant positive correlation reported between satisfaction with selected major and career identity [28], greater satisfaction with the nursing major may strengthen students’ professional identity, thus promoting positive perceptions of their future careers. In other words, students who choose their major based on personal aptitude and interest and experience greater satisfaction with their chosen major are likely to show greater interest in their career, actively engage in career identity formation, and undertake career preparation behaviors, thus positively perceiving the nursing profession. Nursing colleges should enhance students’ understanding of nursing through curricular education and actively implement extracurricular programs, mentoring initiatives, and career vision development activities to broaden their awareness of potential career paths. Conversely, nursing students perceiving greater economic constraints held negative future perceptions regarding securing decent work. Economic constraints are recognized within PWT as predictors affecting perceptions of securing decent work [7]. Similarly, studies conducted among Korean nursing students showed economic constraints influenced work volition, subsequently affecting future perceptions of securing decent work [9]. Economic circumstances are critical factors influencing job choice and perceptions of potential employment. Therefore, universities should proactively implement comprehensive support measures, such as scholarship programs, to assist economically disadvantaged students, thus preventing attrition and reducing career barriers that negatively influence their future employment perceptions.

Correlation analysis among participants’ future perceptions of securing decent work, career adaptability, nursing professionalism, and social comparison orientation showed positive correlations between future perceptions of securing decent work and both career adaptability and nursing professionalism. However, social comparison orientation was not significantly correlated with these variables. Career adaptability, defined as the self-regulation ability to manage career-related issues, is a critical factor influencing perceptions of decent work [7]. Prior domestic studies have also reported that career adaptability indirectly affects future perceptions of securing decent work [29]. Career adaptability was confirmed as relevant for managing nursing students’ future career uncertainties and influencing their occupational decisions. As career adaptability is essential for university students in establishing and achieving occupational goals, proactive exploration of career opportunities should be encouraged through career-related education and clinical practicums, which enable students to indirectly experience the professional world. Additionally, students should enhance career adaptability by proactively recognizing shifts in future employment environments and actively exploring diverse nursing-related career pathways.

This study identified a positive correlation between nursing professionalism and future perceptions of securing decent work. Nursing professionalism influences beliefs and attitudes toward the nursing profession and impacts nursing students’ career identity [30] and nurses’ turnover intentions [31], caring efficacy, and ethical sensitivity. Furthermore, nursing professionalism established during nursing education develops into clinical nursing professionalism [32]. Given that nursing professionalism begins forming through nursing education and continues developing through clinical experience, establishing a solid foundation of nursing professionalism during nursing education is crucial. Since nursing professionalism significantly influences perceptions of securing decent work, universities should actively foster nursing values and career perspectives among students, considering the rapidly changing employment landscape. Although nursing is a highly specialized profession, the social recognition of nursing as a professional field remains somewhat limited [33]. Considering increased coursework and diverse clinical practicum experiences positively influence nursing professionalism formation [33], effective educational programs targeting nursing professionalism should be implemented when third- and fourth-year nursing students experience clinical practice environments.

This study found no significant correlation between social comparison orientation and future perceptions of securing decent work. Although direct comparisons are limited because of the lack of studies specifically investigating social comparison orientation among nursing students, Buunk et al. [34] reported that high levels of social comparison orientation could lead to negative emotions through comparisons with superior others, potentially resulting in nurse burnout. It has also been suggested that perceiving external circumstances as uncertain positively affects social comparison orientation because of low uncertainty tolerance, subsequently increasing employment anxiety [19]. Social comparisons triggered by low uncertainty tolerance negatively influence employment-related outcomes. Although higher employment uncertainty may activate social comparison orientation [12], the lack of a significant correlation between social comparison orientation and future perceptions of securing decent work in this study might have resulted from nursing students traditionally perceiving high employability rates for nurses and considering recent reductions in nurse recruitment due to medical sector conflicts as temporary. Therefore, caution is advised in interpreting these results, as employment market conditions at the time of data collection, such as the sharp decline in nurse recruitment [5], might have influenced the outcomes.

Regression analysis incorporating general characteristics that showed significant differences in future perceptions of securing decent work, as well as career adaptability and nursing professionalism, revealed that motivation for choosing a major, satisfaction with selected major, economic constraints, and nursing professionalism were significant predictors of future perceptions of securing decent work. Although nursing students typically have more concretely developed employment plans than general university students [9], occupational uncertainty negatively impacts their perceptions of future careers and hinders their entry into decent jobs [6]. Research on Genereation Z (Gen Z) university students’ perceptions of decent work indicated that decent jobs are viewed as positions offering above-average wages, guaranteed work-life balance, sustained future growth potential, and high subjective satisfaction [35]. For nursing to be perceived as decent work by Gen Z, subjective and psychological factors, such as guaranteed work-life balance and job satisfaction, must be considered along with tangible improvements in workload and working hours [3], particularly given the rapidly changing societal context. Given that nursing professionalism was identified as a positive influencing factor for future perceptions of securing decent work, increased efforts should be dedicated to cultivating it, especially amid heightened occupational uncertainty.

Although the PWT posits career adaptability as a predictor of decent work [7], it was not identified as a direct influencing factor in this study. This finding aligns with previous domestic studies reporting that career adaptability has a significant indirect, not direct, effect on future perceptions of decent work [29]. Similarly, a study examining the serial multiple mediation effects of work volition and career adaptability on nursing students’ future perceptions of securing decent work demonstrated that career adaptability functions as a mediating rather than an independent variable [9]. Therefore, further replication studies are required to clearly delineate the role of nursing students’ career adaptability in relation to their future perceptions of securing decent work.

This study investigated the factors influencing nursing students’ future perceptions of securing decent jobs after graduating from college. Most nursing students go on to work as nurses after graduation. This study is significant in that it examined these factors based on the PWT at a pivotal time of structural change for the hospital. Alongside discussions about improving traditional nursing roles and working conditions, universities should enhance students’ satisfaction with their major, emphasize education for establishing nursing professionalism, and actively provide diverse economic support measures to students experiencing financial hardships, thus reinforcing perceptions of nursing as a viable and decent career in the face of societal shifts.

Based on the findings, the following recommendations are proposed: First, future studies should extend participant recruitment to encompass diverse geographical areas and all academic years, as this study focused exclusively on third- and fourth-year students from selected nursing colleges. Second, considering the potential impact of external conditions, such as the sharp decline in nurse recruitment at the time of data collection, replication studies are recommended. Lastly, further studies should be conducted among nursing students to verify relationships among additional variables identified within the PWT.

## CONCLUSION

This study employed a descriptive cross-sectional design grounded in the PWT to identify factors influencing nursing students’ future perceptions of securing decent work. The findings confirmed that intrinsic motivation for choosing a major, satisfaction with selected major, economic constraints, and nursing professionalism significantly influenced nursing students’ future perceptions of securing decent work. Given the rapidly changing occupational environment in the digital age, it is critical to understand these dynamics and support nursing students in overcoming career barriers as they prepare to enter the nursing workforce. Therefore, to ensure Gen Z nursing students perceive nursing as decent work in the future, it is essential to enhance infrastructure, including working environments and staffing. Furthermore, universities should emphasize increasing students’ satisfaction with their major, providing financial support to economically disadvantaged students, and reinforcing education to enhance nursing professionalism.

## Figures and Tables

**Table 1.** General Characteristics of Participants (N = 169)

Variables	Categories	n (%)
Sex	Men	34 (20.1)
	Women	135 (79.9)
School year	3rd	69 (40.8)
	4th	100 (59.2)
Motivation for choosing major	Intrinsic	85 (50.3)
	Extrinsic	84 (49.7)
Satisfaction with major	Satisfied	107 (63.3)
	Moderate	50 (29.6)
	Dissatisfied	12 (7.1)
Economic constraints	High	45 (26.6)
	Moderate	47 (27.8)
	Low	77 (45.6)

**Table 2.** Descriptive Findings for Key Variables (N = 169)

Variables	M ± SD	Min	Max	Skewness	Kurtosis
Future perceptions of securing decent work	4.47 ± 0.77	2.53	6.60	0.37	−0.26
Career adaptability	4.01 ± 0.45	3.00	5.00	0.17	0.08
Nursing professionalism	3.96 ± 0.43	3.00	5.00	0.36	−0.38
Social comparison orientation	3.68 ± 0.55	2.20	5.00	−0.30	0.10

M = Mean; SD = Standard deviation; Min = Minimum; Max = Maximum.

**Table 3.** Differences in Future Perceptions of Securing Decent Work According to the Participants’ General Characteristics (N = 169)

Variables	Categories	M ± SD	t or F	*p*
				Scheffe
Sex	Men	4.64 ± 0.68	1.43	.154
	Women	4.43 ± 0.78		
School year	3rd	4.42 ± 0.81	−0.76	.448
	4th	4.51 ± 0.73		
Motivation for choosing major	Intrinsic	4.68 ± 0.75	3.62	< .001
	Extrinsic	4.27 ± 0.73		
Satisfaction with major	Satisfied^a^	4.76 ± 0.67	26.22	< .001
	Moderate^b^	3.98 ± 0.68		a > b,c
	Dissatisfied^c^	4.00 ± 0.71		
Economic constraints	High^a^	4.17 ± 0.71	6.82	.001
	Moderate^b^	4.42 ± 0.68		a < c
	Low^c^	4.68 ± 0.80		

M = Mean; SD = Standard deviation.

**Table 4.** Correlations between Variables (N = 169)

Variables	Future perceptions of securing decent work	Career adaptability	Nursing professionalism
	r (*p*)		
Career adaptability	.35 (< .001)	1	-
Nursing professionalism	.51 (< .001)	.43 (< .001)	1
Social comparison orientation	−.09 (.232)	.03 (.659)	.14 (.081)

**Table 5.** Factors Affecting Future Perceptions of Securing Decent Work (N = 169)

Variables	Categories	Model 1					Model 2				
		B	SE	β	t	*p*	B	SE	β	t	*p*
(Constant)		0.29	0.01		33.40	< .001	0.08	0.04		2.36	.019
Motivation for choosing major	Intrinsic (ref)										
	Extrinsic	−0.28	0.10	−.18	−2.75	.007	−0.25	0.09	−.16	−2.68	.008
Satisfaction with major	Dissatisfied	0.01	0.01	.01	0.07	.948	0.03	0.10	.02	0.26	.799
	Moderate (ref)										
	Satisfied	0.05	0.01	.44	6.13	< .001	0.03	0.01	.27	3.86	< .001
Economic constraints	High	−0.03	0.01	−.26	−3.74	< .001	−0.04	0.01	−.31	−4.94	< .001
	Low (ref)										
	Moderate	−0.01	0.01	−.06	−0.79	.434	−0.01	0.01	−.03	−0.45	.657
Career adaptability							0.12	0.01	.09	1.29	.198
Nursing professionalism							0.82	0.15	.38	5.60	< .001
R^2^ (Adjusted R^2^), F (*p*)		.33 (.31), 15.86 (< .001)					.47 (.45), 20.41 (< .001)				

SE = Standard error; ref= Reference.

## Data Availability

The data are available from the corresponding author upon reasonable request.
